# The Pharmacodynamic Impact of Apremilast, an Oral Phosphodiesterase 4 Inhibitor, on Circulating Levels of Inflammatory Biomarkers in Patients with Psoriatic Arthritis: Substudy Results from a Phase III, Randomized, Placebo-Controlled Trial (PALACE 1)

**DOI:** 10.1155/2015/906349

**Published:** 2015-04-20

**Authors:** Peter H. Schafer, Peng Chen, Lorraine Fang, Andrew Wang, Rajesh Chopra

**Affiliations:** ^1^Translational Development, Celgene Corporation, 86 Morris Avenue, Summit, NJ 07901, USA; ^2^Biostatistics, Celgene Corporation, 33 Technology Drive, Warren, NJ 07059, USA

## Abstract

Apremilast, an oral phosphodiesterase 4 inhibitor, demonstrated effectiveness (versus placebo) for treatment of active psoriatic arthritis in the psoriatic arthritis long-term assessment of clinical efficacy (PALACE) phase III clinical trial program. Pharmacodynamic effects of apremilast on plasma biomarkers associated with inflammation were evaluated in a PALACE 1 substudy. Of 504 patients randomized in PALACE 1, 150 (placebo: *n* = 51; apremilast 20 mg BID: *n* = 51; apremilast 30 mg BID: *n* = 48) provided peripheral blood plasma samples for analysis in a multiplexed cytometric bead array assay measuring 47 proteins associated with systemic inflammatory immune responses. Association between biomarker levels and achievement of 20% improvement from baseline in modified American College of Rheumatology (ACR20) response criteria was assessed by logistic regression. At Week 24, IL-8, TNF-*α*, IL-6, MIP-1*β*, MCP-1, and ferritin were significantly reduced from baseline with apremilast 20 mg BID or 30 mg BID versus placebo. ACR20 response correlated with change in TNF-*α* level with both apremilast doses. At Week 40, IL-17, IL-23, IL-6, and ferritin were significantly decreased and IL-10 and IL-1 receptor antagonists significantly increased with apremilast 30 mg BID versus placebo. In patients with active psoriatic arthritis, apremilast reduced circulating levels of Th1 and Th17 proinflammatory mediators and increased anti-inflammatory mediators.

## 1. Introduction

Psoriatic arthritis (PsA), which occurs in up to 30% of patients with psoriasis, is prevalent in an estimated 0.3% to 1.0% of the general population [1]. Psoriasis and PsA are disease processes driven by overproduction of inflammatory mediators released by innate and adaptive immune cells [2, 3]. Key components of these processes are plasmacytoid dendritic cells, T helper 1 (Th1) cells, and T helper 17 (Th17) cells, which give rise to and maintain the inflammatory cascade [2].

Apremilast, a phosphodiesterase 4 inhibitor (PDE4), helps to regulate the immune response that causes inflammation and skin disease associated with psoriasis and PsA [3–5]. In vitro, apremilast affects production of cytokines and chemokines from peripheral blood mononuclear cells (PBMC) and polymorphonuclear leukocytes, including monocytes, plasmacytoid dendritic cells, T cells, natural killer cells, and neutrophils [5, 6]. Among these effects, the inhibition of tumor necrosis factor (TNF)-*α*, interleukin (IL)-23, and IL-17 production are noteworthy, given the important role of these cytokines in the pathophysiology of psoriatic disease. The production of chemokines by toll-like receptor (TLR)4-stimulated PBMC is also sensitive to PDE4 inhibition, as is TLR2-stimulated neutrophil IL-8 production [5]. Because PDE4 is also expressed in cell types resident in the joints and skin, apremilast also inhibits TNF-*α* production by rheumatoid synovial membranes [7] and keratinocytes in vitro [5]. Many of these preclinical pharmacological observations have been confirmed in clinical pharmacodynamic studies. In the first phase II study of apremilast in psoriasis, treatment with 20 mg QD resulted in a decrease in epidermal thickness, dendritic cell and T-cell skin infiltration, and TNF-*α* production in whole blood ex vivo [8]. Subsequently, in a phase II study in patients with recalcitrant psoriasis, apremilast 20 mg BID led to decreases in proinflammatory gene expression in the lesional skin, including IL-8, IL-12/IL-23p40, IL-17A, and IL-23p19, as well as inducible nitric oxide synthase [9]. In patients with at least a 75% improvement in Psoriasis Area and Severity Index (PASI-75) response, the downregulation of most of these genes was greater than in the nonresponders, yet the expression of IL-10 was increased in responders compared with nonresponders [9]. Therefore, although the local anti-inflammatory effects of apremilast 20 mg had been observed in the lesional skin of psoriasis patients, the effects of the 30 mg BID dose on systemic inflammatory markers had not been explored in psoriatic disease.

The efficacy and safety of apremilast have been evaluated in patients with active PsA in the psoriatic arthritis long-term assessment of clinical efficacy (PALACE) phase III clinical trial program. PALACE 1 compared the efficacy and safety of apremilast with placebo in patients with active PsA despite prior conventional disease-modifying antirheumatic drugs (DMARDs) and/or biologics [10]. In PALACE 1, apremilast demonstrated significant efficacy in improving the signs and symptoms and physical function related to PsA, with sustained responses observed over 52 weeks [10, 11]. In March 2014, the US Food and Drug Administration approved apremilast for the treatment of adults with active PsA, and in September 2014, apremilast was approved for the treatment of patients with moderate to severe plaque psoriasis who are candidates for phototherapy or systemic therapy [12].

In this study, we evaluated the pharmacodynamic effects of apremilast on plasma biomarkers associated with inflammation in a subset of PALACE 1 patients and examined the relationship between change in select biomarkers and PsA clinical response.

## 2. Materials and Methods

### 2.1. Key Inclusion and Exclusion Criteria

Detailed patient selection criteria have been published previously [10]. Briefly, patients were eligible to enroll if they were ≥18 years of age with a ≥6 month history of diagnosed PsA at screening. Patients were required to meet classification criteria for psoriatic arthritis (CASPAR) at study entry and to have three or more swollen and three or more tender joints despite past or current DMARDs and/or biologics, including failures. Patients taking methotrexate, leflunomide, or sulfasalazine must have received stable doses for at least 16 weeks (methotrexate: ≤25 mg/week; leflunomide: ≤20 mg/day; sulfasalazine: ≤2 g/day, or a combination).

Patients with erythrodermic, guttate, or generalized pustular psoriasis, or rheumatic disease other than PsA were excluded. Patients also were excluded if they had active tuberculosis, a history of incompletely treated tuberculosis or significant infection ≤4 weeks of screening (no screening was required for latent tuberculosis), or history of other clinically significant disease or presence of other major uncontrolled disease. Patients could not participate if they had prior therapeutic failure of more than three agents for PsA or more than one TNF blocker.

Patients could continue to take stable doses of DMARDs throughout the trial, as well as stable doses of oral corticosteroids (prednisone ≤10 mg/day or equivalent ≥1 month before screening), nonsteroidal anti-inflammatory drugs, and opioid analgesics (≥2 weeks before screening). Low potency topical corticosteroids, coal tar shampoo and/or salicylic acid scalp preparations, and nonmedicated emollient could be used, except less than 24 hours before each study visit.

### 2.2. Study Design

**Figure 1:**
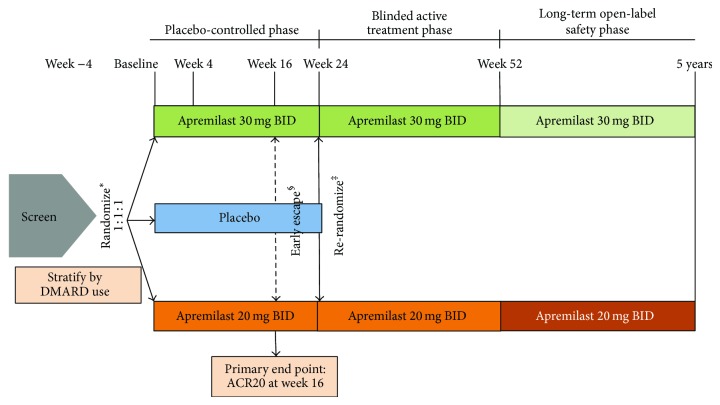
The PALACE 1 study design. Plasma samples for the biomarker assay were obtained at baseline and at Weeks 4, 16, and 24.  ^*^All doses were titrated over the first week of treatment.  ^§^Patients whose swollen and tender joint counts had not improved by ≥20% at Week 16 were considered nonresponders and were required to be rerandomized (1 : 1) to apremilast 20 mg BID or 30 mg BID if they were initially randomized to placebo, or continued on their initial apremilast dose. ^‡^At Week 24, all remaining placebo patients were rerandomized to apremilast 20 mg BID or 30 mg BID.

PALACE 1 was a phase III, multicenter, randomized, double-blind, placebo-controlled study (Figure 1). Eligible patients were randomized (1 : 1 : 1) to receive placebo, apremilast 20 mg BID, or apremilast 30 mg BID for 24 weeks, stratified by baseline DMARD use (yes/no). All doses were titrated over the first week of treatment (10 mg on the first day, increased by 10 mg/day until the target dose was reached). Patients whose swollen and tender joint counts had not improved by ≥20% at Week 16 were considered nonresponders and were required to be rerandomized (1 : 1) to apremilast 20 mg BID or 30 mg BID if they were initially randomized to placebo, or continued on their initial apremilast dose. At Week 24, all remaining placebo patients were rerandomized to apremilast 20 mg BID or 30 mg BID. Upon completion of the 52-week, double-blind period, patients could enter a long-term safety phase.

### 2.3. Biomarker Substudy

A total of 150 patients who were enrolled in PALACE 1 provided written informed consent to participate in the biomarker substudy, which was conducted at 34 centers in the United States and Canada. Patients in the substudy were randomized to double-blind treatment, as specified in the protocol; no additional substudy randomization was performed. Substudy patients were treated exactly as all other PALACE 1 participants, except that blood samples drawn into 6 mL EDTA Vacutainer tubes for the biomarker assay were obtained at baseline and Weeks 4, 16, 24, and 40. The blood samples were processed within 30 minutes of collection by centrifugation at 1200 ×g for 15 minutes at 4°C. Plasma was removed from the blood tubes, transferred as four equal aliquots, each in a 2 mL cryovial tube, frozen on dry ice, and stored at −70°C until analysis.

### 2.4. Pharmacodynamic Assessments

A panel of 47 protein analytes was assessed in patient plasma samples using a validated, multiplexed cytometric bead array immunoassay (Human InflammationMAP 1.0, Myriad RBM, Inc., Austin, TX, USA). The analytes were as follows: alpha-1 antitrypsin, alpha-2 macroglobulin, *β*-2 microglobulin, brain-derived neurotrophic factor, C-reactive protein, complement 3, eotaxin, factor VII, ferritin, fibrinogen, granulocyte-macrophage colony-stimulating factor, haptoglobin, intercellular adhesion molecule-1, interferon-*γ*, IL-1*α*, IL-1*β*, IL-1 receptor antagonist (IL-1RA), IL-2, IL-3, IL-4, IL-5, IL-6, IL-7, IL-8, IL-10, IL-12/IL-23p40, IL-12p70, IL-15, IL-17A, IL-18, IL-23, matrix metalloproteinase (MMP) 2, MMP3, MMP9, macrophage inhibitory protein (MIP)-1*α*, MIP-1*β*, monocyte chemotactic protein (MCP)-1, regulated on activation-normal T-cell expressed and secreted (RANTES), stem cell factor, tissue inhibitor of metalloproteinase, TNF-*α*, TNF-*β*, TNF-*α*2, vascular cellular adhesion molecule type 1, vascular endothelial growth factor, von Willebrand factor (vWF), and vitamin D binding protein.

Clinical response was assessed based on achievement of 20% improvement from baseline in modified American College of Rheumatology (ACR20) response criteria [13]. ACR20 response at Week 16 was the primary efficacy end point in PALACE 1.

### 2.5. Statistical Analysis

Analyses were performed in biomarker substudy patients who were randomized, received ≥1 dose of study medication, and had ≥1 baseline and ≥1 postbaseline value for any biomarker. Analyses were conducted for datasets derived from the placebo-controlled period (Weeks 0 to 24) and the apremilast-exposure period (Weeks 0 to 52). For the placebo-controlled period, the change from baseline for each biomarker at each sampled time point was compared between each apremilast dose (20 mg BID and 30 mg BID) and placebo using a nonparametric, rank analysis of covariance model, with treatment group as a factor and baseline biomarker value as a covariate. For Weeks 16 and 24, missing values were imputed using the last-observation-carried-forward (LOCF) methodology; for Week 4, the analysis was conducted based on observed data. Patients initially randomized to placebo who were rerandomized to apremilast at Week 16 were not included in between-treatment comparisons for Week 24. *P* values were not adjusted for multiplicity and were used only as measures of strength of association. Because the 40-week apremilast-exposure period lacked a placebo-control group, within-treatment biomarker change from baseline (the last value measured on or before the date of first apremilast dose) was examined based on observed data using a two-sided Wilcoxon signed rank test. Data for patients initially randomized to placebo and later rerandomized to either apremilast dose were analyzed according to the actual number of weeks each of these patients received apremilast treatment. Because of this, biomarker data over 40 weeks are expressed in terms of weeks of apremilast exposure, rather than by study week. Odds ratios (ORs) and 95% confidence intervals (CIs) were also calculated.

To describe the relationship between ACR20 response and change from baseline in biomarkers, those biomarkers that exhibited significant change from baseline (*P* < 0.05) with apremilast 20 mg BID or 30 mg BID compared with placebo at Week 16 (LOCF) or Week 24 (LOCF) were included in univariate and multivariate regression analyses, with ACR20 response as the dependent variable and biomarker change as the covariate. The multivariate model included terms for treatment and biomarker change by treatment interaction. Missing data were handled using the nonresponder imputation rule, LOCF approach (ACR20 response), or LOCF approach only (biomarker data).

## 3. Results

A total of 150 patients (29.8% of the 504 patients comprising the full study population) provided blood samples for biomarker analysis (placebo: *n* = 51; apremilast 20 mg BID: *n* = 51; and apremilast 30 mg BID: *n* = 48). Among substudy patients, baseline demographic and disease characteristics, as well as prior and concurrent therapy, were comparable across treatment groups (Table 1). Baseline demographics were similar to those of the full study population, except that prior exposure to methotrexate was higher in the biomarker subset (80.0%), as was prior exposure to a biologic DMARD, such as a TNF blocker (48.8%), than in the overall intent-to-treat population (54.2% and 23.6%, resp.) (Table 1). Prior biologic failure was also higher in the biomarker subset (20.7%) than in the overall intent-to-treat population (9.3%) (Table 1). The biomarker substudy patients, therefore, may have had a more treatment-resistant PsA phenotype than the overall study population.

### 3.1. Biomarker Changes during the Placebo-Controlled Period (Weeks 0 to 24)

**Figure 2:**
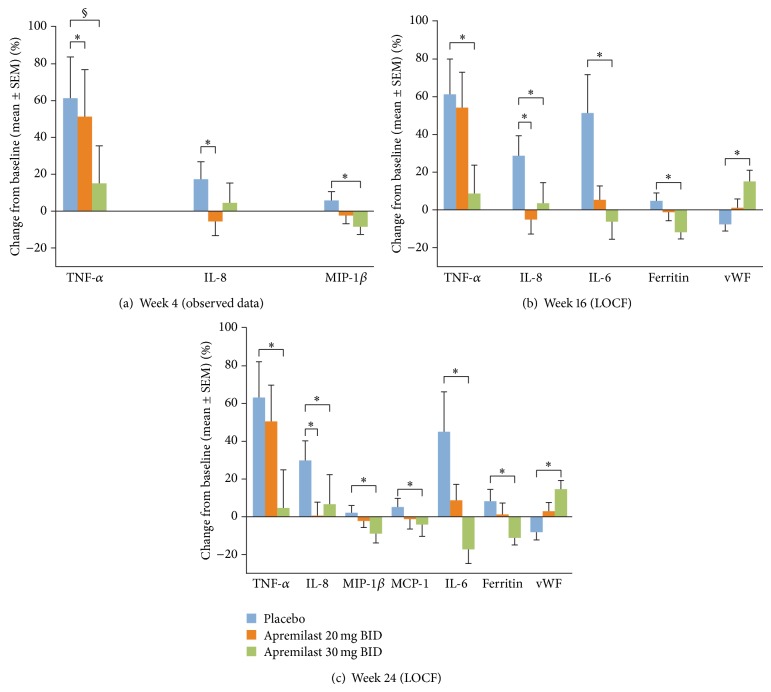
Changes in biomarkers with placebo, apremilast 20 mg BID, and apremilast 30 mg BID over Weeks 0 to 24.  ^*^ *P* < 0.05 versus placebo (rank analysis of covariance two-sided *P* value).  ^§^ *P* = 0.0527. SEM: standard error mean.

Patients who received apremilast 20 mg BID or 30 mg BID exhibited significantly different mean percent changes from baseline in various biomarkers at Weeks 4, 16, and/or 24 compared with placebo (Figure 2). At Week 16 (LOCF), with apremilast 20 mg BID and/or apremilast 30 mg BID treatment, significant differences in mean percent change from baseline (versus placebo) were observed for TNF-*α*, IL-8, IL-6, and ferritin. At Week 24 (LOCF), apremilast 20 mg BID or 30 mg BID treatment was associated with significantly different percent changes from baseline (versus placebo) in TNF-*α*, IL-8, IL-6, ferritin, MIP-1*β*, and MCP-1 (Figure 2). Significant mean percent increases (versus placebo) in vWF were also observed with apremilast treatment at Weeks 16 and 24 (Figure 2); however, all vWF values were within normal range (<120 *μ*g/mL).

### 3.2. Association between ACR20 Response and Change in Biomarkers

At Week 16, changes in plasma TNF-*α* and vWF were associated with ACR20 response based on both univariate and multivariate logistic regression analyses (Table 2). For TNF-*α*, the association with ACR20 response was significant for both apremilast dose groups. ACR20 response was associated with an increase from baseline in TNF-*α* in patients receiving apremilast 20 mg BID but a decrease from baseline in TNF-*α* in patients receiving apremilast 30 mg BID. In the apremilast 20 mg BID group, there was a mean 120% increase from baseline TNF-*α* at Week 16 among the ACR20 responders, with a high degree of variability (data not shown). By contrast, in the apremilast 30 mg BID dose group, there was a mean 40% decrease from baseline TNF-*α* at Week 16 among the ACR20 responders. For vWF, the association with ACR20 response was significant only for the apremilast 20 mg BID group.

### 3.3. Change in Biomarkers over 40 Weeks

**Figure 3:**
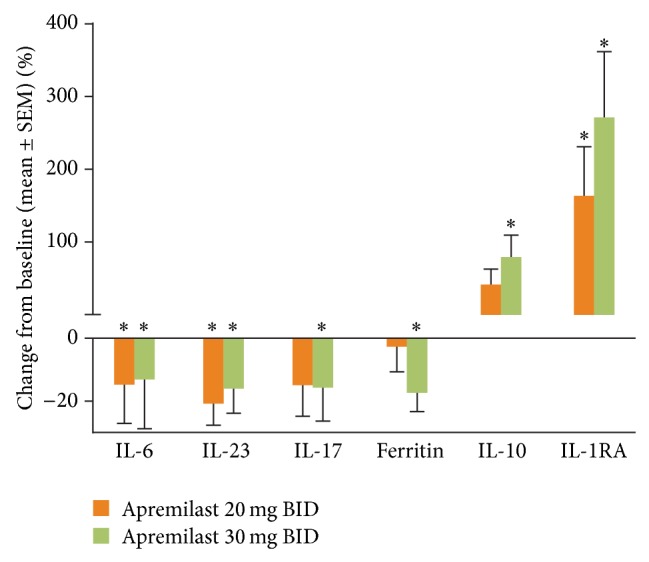
Mean percent change in biomarkers with apremilast 20 mg BID and apremilast 30 mg BID at Week 40; no patients were receiving placebo at this time point. ^*^ *P* < 0.05 Wilcoxon signed rank test (two-sided *P* value for testing the median of zero).

**Figure 4:**
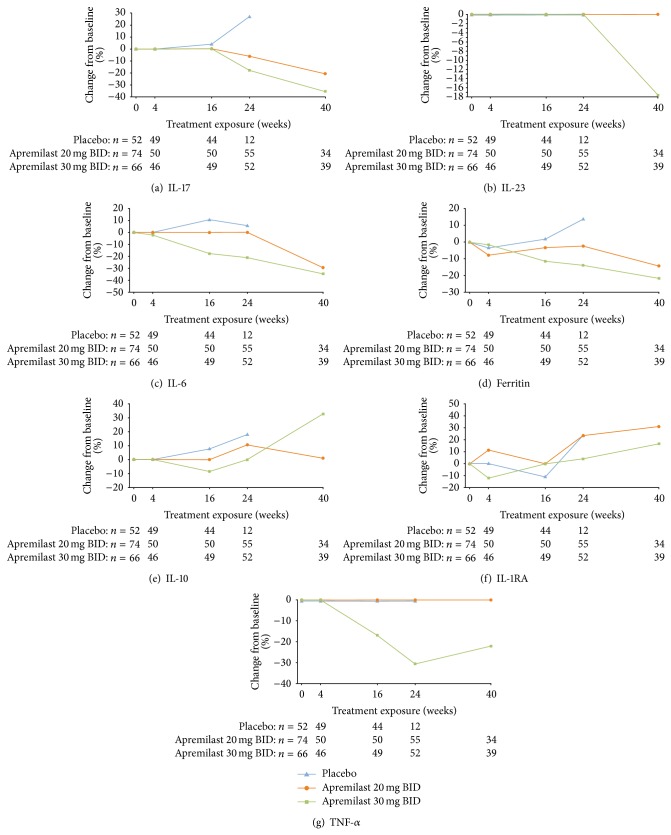
Median percent change in select biomarkers with placebo, apremilast 20 mg BID, and apremilast 30 mg BID over Weeks 0 to 40.

Six biomarkers (IL-6, IL-17, IL-23, ferritin, IL-10, and IL-1RA) exhibited significant changes (versus baseline) at Week 40 among patients who received apremilast 20 mg BID or 30 mg BID (Figure 3). At this time point, there was no longer any placebo-treated group for comparison, because all placebo-treated subjects were rerandomized to receive apremilast 20 mg BID or 30 mg BID at Week 16 (early escape) or Week 24 (the final visit for the placebo-controlled portion of the study). With apremilast treatment, the biomarker change from baseline varied over time. For some analytes, differential effects of apremilast 20 mg BID and 30 mg BID doses were observed (Figures 4(a)–4(f)). Changes in biomarkers that were maintained over time to Week 40 with apremilast 20 mg BID and/or 30 mg BID included, but were not limited to, decreases in IL-17, IL-6, and ferritin, and increases in IL-10 and IL-1RA (Figure 4).

## 4. Discussion

The current exploratory pharmacodynamic findings in patients with PsA enrolled in the PALACE 1 trial substudy support previous reports surrounding the biological activity of apremilast on immune cell function and in psoriatic disease [6, 9]. Patients receiving apremilast over 24 weeks exhibited significant changes in multiple inflammatory biomarkers, including IL-8, TNF-*α*, IL-6, MIP-1*β*, and MCP-1, consistent with the anticipated broad effect of PDE4 inhibition [5]. Changes in TNF-*α* with apremilast 20 mg BID and 30 mg BID treatment were associated with ACR20 clinical response at Week 16, the primary end point. After 40 weeks of apremilast treatment, generally similar decreases in the plasma concentration of IL-6, IL-17, and IL-23 were seen with both apremilast doses, suggesting long-term inhibition of the systemic Th-17 immune response. The significant increases in IL-10 and IL-1RA after 40 weeks of apremilast treatment demonstrate the increase in anti-inflammatory mediator production with PDE4 inhibition.

The profile of biologic activity observed is consistent with a therapeutic role for apremilast in the treatment of PsA. Many of the inflammatory analytes down-regulated during apremilast treatment in the current subset of PALACE 1 patients, including TNF-*α*, IL-8, IL-17, MIP-1*β*, and MCP-1, are over-expressed in patients with psoriasis and PsA [14–17]. The current findings are largely consistent with preclinical studies of the impact of apremilast on cytokine and chemokine expression [5, 6]. For Weeks 4 through 24, biomarker changes were generally greater in the apremilast 30 mg BID treatment group than in the apremilast 20 mg BID treatment group (Figure 2). Interestingly, at Week 40 the magnitude of the effects on IL-6, IL-23, and IL-17 were similar for the apremilast 20 mg BID and 30 mg BID treatment groups (Figure 3). This suggests that the clinical pharmacodynamics of the two apremilast dose levels became similar over the longer treatment duration. In the overall study population, clinical efficacy at Week 40 for patients initially randomized to apremilast at baseline was also similar for the apremilast 20 mg BID and 30 mg BID treatment groups, with ACR20 response rates of 58% for apremilast 20 mg BID and 57% for apremilast 30 mg BID [18]. Previously, in a phase II study in patients with recalcitrant psoriasis, treatment with apremilast 20 mg BID led to significant decreases in proinflammatory gene expression in the lesional skin, including IL-12/IL-23p40, IL-17A, and IL-23p19 [9]. Taken together, these results illustrate that apremilast has a significant impact on the Th-17 mediators in both the systemic (blood) and local (skin) immune compartments. In psoriatic disease, IL-17 is produced not only by T helper cells, but also neutrophils, mast cells, *γδ* T cells, and innate lymphoid cells [19]. In addition, in psoriasis, keratinocytes can produce IL-17C, and such production is associated with psoriatic skin inflammation [20]. Whether apremilast has direct or indirect effects on IL-17 production by all of these cell types is not yet known. It should be noted that only IL-17A, and not other isoforms such as IL-17C or IL-17F, was measured in the current study. In an earlier study of apremilast, decreases in IL-17A were linked to decreased keratinocyte proliferation and to PASI-75 response in patients with recalcitrant psoriasis [9]. Moreover, in a recent phase III trial, the human anti-IL-17A monoclonal antibody secukinumab demonstrated efficacy for PsA [21]. The current findings build on this evidence and suggest a potential role of IL-17 reduction in the clinical response to apremilast treatment of PsA; it will be of interest to explore this relationship in future investigations. Interestingly, IL-10 levels were significantly increased after 40 weeks of treatment with apremilast 30 mg BID. This may have been due to a direct induction of IL-10 by apremilast, as had been observed, to elevate IL-10 production by endotoxin-stimulated PBMC, and in the skin of psoriasis patients who achieved a clinical response PASI-75 or greater [5, 9].

However, there were some unexpected observations. Preclinical investigations indicated that apremilast leads to increased expression of IL-6 at higher concentrations in vitro (e.g., 10 *μ*M) [5]. In this study, however, IL-6 plasma levels were significantly decreased by apremilast 30 mg BID at Weeks 16, 24, and 40. This decrease in IL-6 may be an indirect effect, caused by the reduction of TNF-*α*, which affects IL-6 expression [22]. In a study of infliximab for PsA, IL-6 also showed early decreases from baseline, although IL-6 levels rebounded somewhat at 12 weeks [23]. In the current study, there was also a significant decline in ferritin in patients treated with apremilast 30 mg BID for 16, 24, and 40 weeks. While the pathophysiological role of ferritin in PsA is not clear, a similar, significant 12.6% median decrease in ferritin has been reported in psoriasis patients treated with etanercept for 12 weeks [24].

Significant increases from baseline in vWF were seen with apremilast 30 mg BID at Week 16 and Week 24. The mechanism by which apremilast increased vWF levels is unknown, although it may be related to apremilast-mediated cyclic adenosine monophosphate (cAMP) elevation within endothelial cells. The vWF is released from Weibel-Palade bodies upon stimulation of endothelial cells with cAMP-elevating secretagogues such as epinephrine, thrombin, histamine, vasopressin, and adenosine, as well as the nonselective phosphodiesterase inhibitor IBMX [25, 26]. Importantly, such vWF elevations remained below the established upper limit of normal (<120 *μ*g/mL) and were transient, returning to baseline levels with up to 40 weeks of treatment.

Regression analyses showed that ACR20 response was associated with changes in TNF-*α* and vWF. At Week 16, ACR20 response was associated with a decrease in TNF-*α* in the apremilast 30 mg BID group; in this dose group, there was a mean 40% decrease from baseline TNF-*α* at Week 16 among the ACR20 responders. However, ACR20 response also was associated with an increase in TNF-*α* with apremilast 20 mg BID; in this dose group, there was a mean 120% increase from baseline TNF-*α* at Week 16 among the ACR20 responders, with a high degree of variability (data not shown). vWF was increased at Week 16 in both apremilast dose groups. The precise reason for this difference in TNF-*α* is not known, but the anti-inflammatory effects of apremilast 30 mg BID at Week 16 appear to be more clear and consistent than the effects of apremilast 20 mg BID. At Week 24, the association between TNF-*α* and ACR20 response was no longer evident, and the association with vWF persisted. While the link between elevated TNF-*α* levels and relatively greater PsA disease activity is fairly well established [17], evidence regarding the clinical significance of vWF changes is conflicting and not well characterized [27, 28]. vWF expression has previously been shown to be elevated in patients with active PsA, as well as other arthropathies [27, 28]. Given the broad nature of the changes among inflammatory mediators observed currently with apremilast treatment, interpreting the relationship between clinical response and single protein analytes must be undertaken with caution. With continued or long-term treatment, early changes in one inflammatory mediator may induce changes in the expression of other, downstream signals; clinical responses may evolve in both magnitude and nature over time. In this context, it seems more plausible that the aggregate of changes in inflammatory mediator expression seen with apremilast over time, rather any single mediator at any one time point, contributes to the clinical effects observed.

The subgroup of patients included in the current pharmacodynamic analysis was limited in size, representing approximately one-third of the overall study population. Proportions of patients with prior exposure to methotrexate and/or to a biologic DMARD, such as a TNF blocker, and the prior biologic failure rate were higher in the biomarker subset than in the overall intent-to-treat population. These differences suggest that the biomarker substudy population may have had a more treatment-resistant PsA phenotype than the overall study population, thereby limiting the extrapolation of the biomarker study conclusions. These differences in prior treatment may have led to an underestimation of the pharmacodynamic effects of apremilast and dampened the association with clinical response, since the PsA patients with previous biologic treatment had lower clinical response rates than the biologic-naïve patients [10]. Fluctuations in disease activity accompanying flares of skin or joint symptoms during the study period within individual patients may also have contributed to variability in cytokine levels. The groups were not matched in terms of ongoing, concomitant DMARD treatment during the study period, which may also have impacted observed differences in biomarker levels.

Similar biomarker studies have been conducted with other therapeutic agents used for the treatment of PsA. For example, treatment of PsA patients with the human TNF-*α* monoclonal antibody golimumab for 4 to 14 weeks was associated with a significant decrease in serum levels of the acute phase reactants serum amyloid P, haptoglobin, and *α*_1_ anti-trypsin, and inflammatory markers MCP-1, MIP-1*β*, IL-16, IL-8, S100A12 intercellular adhesion molecule 1, MMP-3, and vascular endothelial growth factor, as well as plasminogen activator inhibitor-1 and thyroxine binding globulin [29]. This pattern for golimumab is only partially overlapping with that of apremilast, namely with respect to the reductions in MCP-1, MIP-1*β*, and IL-8 observed for both therapeutic agents. However, the remainder of the biomarker changes were quite distinct. In another example, infliximab therapy was associated with significant decreases in serum IL-6, vascular endothelial growth factor, FGF, MMP-2, and E-selectin early after infliximab infusions (2–6 weeks), and TNF-*α* levels decreased after 12 weeks in a small cohort of PsA patients [23]. Here, there were similarities between the mechanisms of infliximab and apremilast in that IL-6 and TNF-*α* were decreased following treatment with either agent. In a small study analyzing the changes in serum biomarkers in PsA patients during treatment with etanercept, adalimumab, golimumab, or infliximab, decreases in MMP-3 and an increase in cartilage oligomeric matrix protein were associated with clinical response to these TNF inhibitors [30]. Such results are quite different from the patterns identified in the current PsA study with apremilast. However, differences in the matrix (serum versus plasma), sample handling, and storage conditions as well as biomarkers, assay methodologies, and PsA patient populations would preclude any solid conclusions regarding a direct comparison between apremilast and these other effective agents.

## 5. Conclusion

Overall, the effects of apremilast in PsA are consistent with its pharmacological profile observed in vitro [6], and these clinical pharmacodynamic data demonstrate that apremilast may exert its therapeutic effects through regulating production of Th1 cytokines and chemokines in the earlier stage of treatment (4–24 weeks), followed by regulation of Th17-related immunity and upregulation of anti-inflammatory mediators upon longer treatment (40 weeks).

## Figures and Tables

**Table 1:** Baseline demographic and clinical characteristics of biomarker substudy patients (*N* = 150) and the overall intent-to-treat population (*N* = 504) [10].

	Biomarker substudy population (*N* = 150)			Overall intent-to-treat population (*N* = 504)^*^		
	Placebo *n* = 51	Apremilast		Placebo *n* = 168	Apremilast	
		20 mg BID *n* = 51	30 mg BID *n* = 48		20 mg BID *n* = 168	30 mg BID *n* = 168
Age, mean (SD), years	49.7 (12.4)	47.3 (11.2)	52.3 (11.2)	51.1 (12.1)	48.7 (11.0)	51.4 (11.7)
Female, *n* (%)	20 (39.2)	25 (49.0)	23 (47.9)	80 (47.6)	83 (49.4)	92 (54.8)
White, *n* (%)	49 (96.1)	46 (90.2)	47 (97.9)	153 (91.1)	150 (89.3)	152 (90.5)
Body mass index, mean (SD), kg/m^2^	30.7 (7.4)	33.0 (7.1)	32.8 (6.8)	31.1 (6.6)	30.9 (7.3)	30.6 (5.9)
Duration, mean (SD), years						
PsA	6.5 (5.7)	4.9 (4.6)	10.1 (8.3)	7.3 (7.1)	7.2 (6.8)	8.1 (8.1)
Psoriasis	13.5 (11.7)	13.1 (11.9)	19.0 (13.5)	15.7 (13.0)	15.5 (11.9)	16.5 (12.3)
Swollen joint count (0–76), mean (SD)	13.0 (8.2)	12.7 (10.4)	13.9 (8.7)	12.8 (8.8)	12.5 (9.5)	12.8 (7.8)
Tender joint count (0–78), mean (SD)	27.9 (17.8)	22.4 (15.6)	27.4 (16.5)	23.3 (15.2)	22.2 (15.9)	23.1 (14.5)
HAQ-DI (0–3), mean (SD)	1.1 (0.59)	1.0 (0.55)	1.2 (0.61)	1.2 (0.6)	1.2 (0.6)	1.2 (0.6)
Physician's global assessment of disease activity (0–100 mm VAS), mean (SD)	58.0 (20.4)	59.4 (18.9)	57.9 (17.4)	55.2 (20.3)	54.1 (21.8)	55.7 (19.2)

Prior use of biologics, *n* (%)	24 (47.1)	24 (47.1)	25 (52.1)	41 (24.4)	37 (22.0)	41 (24.4)
Prior use of methotrexate, *n* (%)	41 (80.4)	40 (78.4)	39 (81.3)	90 (53.6)	95 (56.5)	88 (52.4)
Prior biologic failure, *n* (%)	11 (21.6)	10 (19.6)	10 (20.8)	19 (11.3)	14 (8.3)	14 (8.3)

^*^ *n* reflects the number of patients randomized; actual number of patients available for each end point may vary.

HAQ-DI: Health Assessment Questionnaire-Disability Index; PsA: psoriatic arthritis; VAS: visual analog scale.

**Table 2:** Association between ACR20 response and biomarker change at Week 16.

	Univariate analysis 1^*^						Multivariate analysis 2^§^
Biomarker^§^	Placebo *n* = 51		Apremilast 20 mg BID *n* = 51		Apremilast 30 mg BID *n* = 48		Interaction
	OR (two-sided 95% CI)	*P* value^‡^	OR (two-sided 95% CI)	*P* value^‡^	OR (two-sided 95% CI)	*P* value^‡^	*P* value
TNF-*α* (pg/mL)	0.995 (0.986, 1.003)	NS	1.006 (1.001, 1.011)	0.0205	0.978 (0.960, 0.996)	0.0166	0.0024

IL-8 (pg/mL)	1.000 (0.990, 1.010)	NS	0.997 (0.985, 1.008)	NS	0.994 (0.984, 1.004)	NS	NS

IL-6 (pg/mL)	0.998 (0.992, 1.004)	NS	1.002 (0.991, 1.013)	NS	0.989 (0.977, 1.002)	NS	NS

Ferritin (ng/mL)	0.996 (0.974, 1.019)	NS	1.001 (0.984, 1.018)	NS	0.992 (0.969, 1.016)	NS	NS

vWF factor (*μ*g/mL)	1.012 (0.985, 1.040)	NS	0.996 (0.938, 0.996)	0.0253	1.007 (0.992, 1.022)	NS	0.0387

NS: not significant.

^*^ORs, CIs, and *P* values were calculated from a logistic regression model with percent change from baseline biomarker value at Week 16 (LOCF) as a covariate.

^§^At Week 16 (LOCF) for the biomarkers with a significantly (*P* < 0.05) different percent change from baseline in the between-treatment comparisons (apremilast 20 mg BID versus placebo or apremilast 30 mg BID versus placebo), the ACR20 (nonresponder imputation) and ACR20 (LOCF) datasets were identical.

^‡^ *P* values were calculated from a logistic regression model with treatment as a factor, percent change from baseline biomarker value at Week 16 (LOCF) as a covariate, and interaction of treatment and percent change from baseline value at Week 16 (LOCF).
